# Salivarian Trypanosomosis: A Review of Parasites Involved, Their Global Distribution and Their Interaction With the Innate and Adaptive Mammalian Host Immune System

**DOI:** 10.3389/fimmu.2018.02253

**Published:** 2018-10-02

**Authors:** Magdalena Radwanska, Nick Vereecke, Violette Deleeuw, Joar Pinto, Stefan Magez

**Affiliations:** ^1^Laboratory for Biomedical Research, Ghent University Global Campus, Incheon, South Korea; ^2^Laboratory of Cellular and Molecular Immunology, Vrije Universiteit Brussel, Brussels, Belgium

**Keywords:** trypanosomosis, immunology, pathology, anemia, transmission

## Abstract

Salivarian trypanosomes are single cell extracellular parasites that cause infections in a wide range of hosts. Most pathogenic infections worldwide are caused by one of four major species of trypanosomes including (i) *Trypanosoma brucei* and the human infective subspecies *T. b. gambiense* and *T. b. rhodesiense*, (ii) *Trypanosoma evansi* and *T. equiperdum*, (iii) *Trypanosoma congolense* and (iv) *Trypanosoma vivax*. Infections with these parasites are marked by excessive immune dysfunction and immunopathology, both related to prolonged inflammatory host immune responses. Here we review the classification and global distribution of these parasites, highlight the adaptation of human infective trypanosomes that allow them to survive innate defense molecules unique to man, gorilla, and baboon serum and refer to the discovery of sexual reproduction of trypanosomes in the tsetse vector. With respect to the immunology of mammalian host-parasite interactions, the review highlights recent findings with respect to the B cell destruction capacity of trypanosomes and the role of T cells in the governance of infection control. Understanding infection-associated dysfunction and regulation of both these immune compartments is crucial to explain the continued failures of anti-trypanosome vaccine developments as well as the lack of any field-applicable vaccine based anti-trypanosomosis intervention strategy. Finally, the link between infection-associated inflammation and trypanosomosis induced anemia is covered in the context of both livestock and human infections.

## Introduction

Human African Trypanosomosis and Animal African Trypanosomosis are two well-known diseases that affect sub-Saharan Africa and have historically prevented the development of vast lands of the African continent into highly productive agricultural areas. However, the first salivarian pathogenic trypanosome to be discovered was *T. evansi*, a parasite identified by Dr. Griffith Evans in 1880, in horses and camels suffering from a disease called Surra on the Indian subcontinent (1). Almost 140 years after this initial discovery, a wealth of world-wide epidemiological data on pathogenic trypanosomes shows they are present on four different continents. Molecular parasite mechanisms, that allow the escape from the hosts' immune and non-immune defense systems, have been discovered and various interactions in the context of vector biology have been described. However, in the end the data available today has still not given us a way to intervene in trypanosomosis transmission by means of an effective anti-parasite vaccination strategy. Hence, control still relies on a combination of active case diagnosis and treatment, as well as vector control (2, 3). In this review we cover the classification of trypanosomes, which has recently become under scrutiny (4), as well as new discoveries with respect to genetic exchange between trypanosomes that takes place in the insect vector (5, 6). In addition, the paper provides an update on recent discoveries with respect to the B cell destructive potential of trypanosomes (7, 8), T cell biology (9), and the impact of trypanosomosis on red blood cell (RBC) homeostasis and infection-associated anemia (10). Throughout the data review, both animal trypanosomosis (AT) and human trypanosomosis (HT) have been considered. However, as most recent data shows, this “artificial” distinction might be less useful than previously thought, as atypical human trypanosomosis (a-HT), which can be caused by various animal trypanosomes, is now gaining more and more attention in the field (11).

## Setting the scene for salivarian trypanosomosis

Trypanosomes are unicellular protozoan organisms of the class Kinetoplastida that cause a wide range of infections in a broad range of hosts. The latter includes not just mammals but also fish (12), birds (13), and reptiles (14), while insect vectors actually should be considered not just as transmission “tools” but also as definite hosts. Indeed, it is only here that sexual reproduction stages have been reported, as comprehensively outlined in a recent review by Gibson W. (5). In mammals, both salivarian and stercorarian trypanosomes cause diseases that affect the health status of the infected host in multiple ways. While the stercorarian trypanosomes are an important group of parasites, the main focus of this review is directed toward the pathogenic salivarian trypanosomes that cause infections in human, livestock, and game animals. These infections are marked by the extracellular nature of the infecting agent, causing pathologies and health complications that are very different from the features that characterize intracellular pathogenic infections such as those caused by the stercorarian *T. cruzi* parasite. An additional complication that arises when describing trypanosomosis, is the use of the term *African Trypanosomosis*. This denomination is very often used in an incorrect way. Indeed, as will be described in this review, all major pathogenic salivarian trypanosome infections do occur on the African continent. However several of the pathogens responsible for these diseases have moved “out of Africa” and infections are progressing throughout the world. A last introductory remark for this paper is the fact that Human African Trypanosomosis or HAT has recently been brought under control in a very significant manner by huge consorted international efforts of the last decennium (15). Hence, this might give the impression that trypanosomosis has become a disease of the past. This however could very well be a wrong assumption for three main reasons. First, there are no reports that suggest that AT is near to being controlled on a world-wide scale. Second, the most aggressive form of HAT caused by *T. b. rhodesiense* has a zoonotic origin, so as long as human infective trypanosomes are present in a wildlife reservoir, re-emergence of the disease remains a risk (16, 17). This holds true even if the majority of infections caused by *T. b gambiense* are being brought under control. Third, reports of a-HT in- and outside Africa show that “African” trypanosomosis is only part of the problem (11). Hence, for now trypanosome diseases still remain a threat to human health and to agriculture systems of emerging economies. In the absence of any vaccine strategy preventing the spread of these infections, continued research into host-parasite interactions is needed. This will provide a better understanding of trypanosome diseases itself, the mechanisms of disease resistance, modes of immune evasion, and ultimately the reasons for continued failure of vaccination attempts.

## Classification of the main pathogenic salivarian trypanosomes

**Figure 1:**
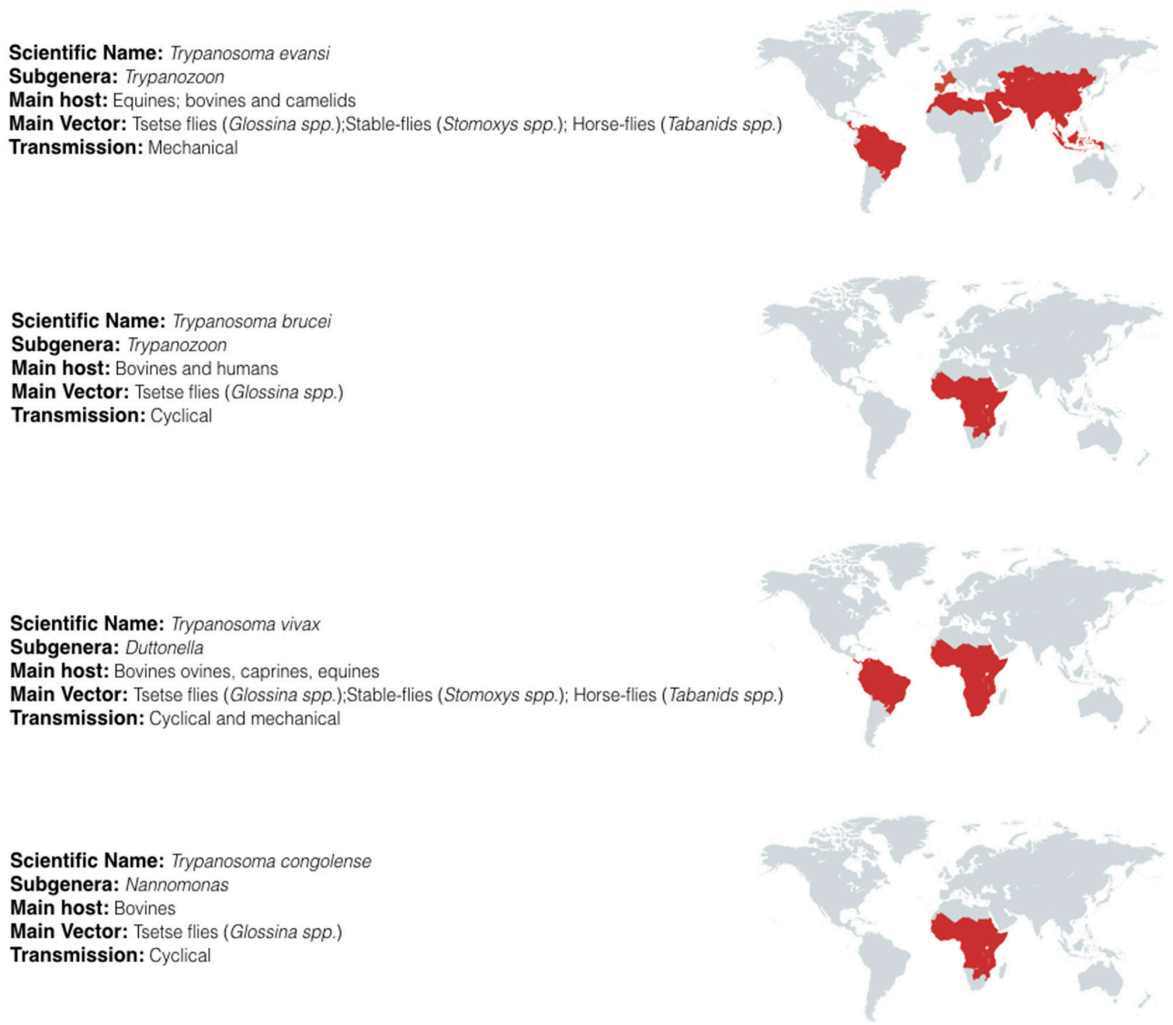
Geographic distribution of salivarian trypanosomosis. Salivarian trypanosomosis is a worldwide problem caused in large by *Trypanosoma evansi, Trypanosoma brucei* (including the human infective subspecies *T. b. gambiense* and *T. b. rhodesiense*), *Trypanosoma vivax* and *Trypanosoma congolense*. *T. brucei*, and *T. congolense* infections are limited to the sub-Saharan tsetse belt. In contrast, as *T. vivax* and *T. evansi* can be mechanically transmitted, these parasites have migrate beyond the tsetse belt, out of Africa and into South America and Asia [adapted from (18–22)].

Trypanosomes belong to the sub-kingdom Protozoa, the order Kinetoplastida, the family Trypanosomatidae, and genus *Trypanosoma*. The large numbers of different species belonging to this genus have been classified in several subgenera according to their morphology. For the salivarian pathogenic trypanosomes the subgenera include *Trypanozoon, Duttonella, Nannomonas*, and *Pycnomonas*, of which the first three account for the vast majority of human and animal infections and are the subject of this review. Their combined geographic spread covers most of the developing world (Figure 1).

The first trypanosome subgenus, *Trypanozoon*, is composed of several *Trypanosoma* species, which are human and animal infective and includes the first pathogenic trypanosome ever to be discovered i.e. *Trypanosoma evansi*. Today, *T. evansi* is a parasite that is considered to have mainly a veterinary importance (1), causing the disease Surra in a wide range of economically important mammals such as horses, cattle, goats, buffalos, dogs, and camels. In addition, the parasite can be found in game animals such as deer, wild pigs, and capybaras, representing a reservoir that often might escape attention. Today, *T. evansi* is found across Central and South America, North Africa, the Russian territories, the Indian subcontinent, China, and Southeast Asia (23). Transmission mainly occurs mechanically through the bite of bloodsucking insects from the family Tabanidae (genus *Tabanus*) (24), Chrysops (25), Atylotus (26), and Muscidae (genus *Stomoxys* and *Haematobia*) (18). It is this mechanical transmission that has allowed the parasite to move beyond the tsetse fly region and out of Africa. Morphologically, *T. evansi* has long been considered as a monomorphic parasite with the main bloodstream form appearing as so called “long slender” forms. However, the appearance of short intermediate forms has also been reported, but only in blood smears of infected cat and monkey (27). Important is that the parasite has a peculiar kinetoplast, characterized by either a reduced (lack of maxi-circles and homogenous mini-circles) or a total absence of kinetoplast DNA (kDNA) (28). This deficiency is thought to lock *T. evansi* in the bloodstream form, as they are unable to transcribe the kDNA genes required to perform the oxidative phosphorylation required for the developmental processes in the midgut of the tsetse (29). For long, this altered kDNA characteristic has been used to differentiate *T. evansi* from African *T. brucei* subspecies. Recently however, genetic analysis of a large battery of both *T. evansi* and *T. brucei* parasites has shown that the situation is more complex, and that many *T. evansi* parasites are closely related to *T. brucei*, even more closely than the relation between *T. evansi* parasites from different geographic locations (30–32). In addition, these data suggest that *T. evansi* arose multiple times from a different *T. brucei* ancestor. Hence, this has sparked a debate about the nomenclature of the *Trypanozoon* parasites. While it has been suggested by some to consider *T. evansi* as a *T. brucei* variant, a most recent revision has been proposed based on the proper application of the principles of biological nomenclature. This proposal suggests to rename all *T. brucei* subspecies as *T. evansi* subspecies, and even adopt the use of *T. evansi gambiense* and *T. evansi rhodesiense* for human infective African Trypanosomes (4). Important in the context of the parasite-host interplay of *T. evansi* is the notion that several human *T. evansi* infections have been reported in- and outside Africa (11). However, despite the wide geographic distribution of *T. evansi*, the reports on human non-African trypanosomosis are overall extremely rare. However, it cannot be excluded that one of the reasons for the scarce amount of data on a-HT is simply due to the lack of proper diagnostic practices that are able to correctly identify human trypanosomosis in *T. evansi* endemic areas.

The second *Trypanozoon* subspecies to be discovered was *T. brucei*, endemic to sub-Saharan Africa and transmitted by biting flies of the genus *Glossina*, commonly known as tsetse, with tsetse meaning “fly” in the Tswana language of Southern Africa. *T. brucei* parasites present a major health problem for humans, as the causative agent of the disease called HAT, or sleeping sickness. Domestic animals such as cattle, pigs, small ruminants, and game animals are also common hosts for *T. brucei*, in which the latter serve as a natural reservoir of the parasite. Three morphological indistinguishable subspecies of *T. brucei* are known (namely *T. b. gambiense, T. b. rhodesiense*, and *T. b. brucei.)* with *T. b. gambiense* and *T. b. rhodesiense* responsible for human trypanosome diseases in West/Central and East Africa, respectively. Uganda is one of the only countries where the two forms of HAT appear in adjacent regions. *T. b. brucei*, on the other hand, is unable to infect humans and is responsible for animal trypanosomosis only. The correct identification of the *T. brucei* subspecies is nearly impossible when solely based on their morphology or geographical origin. Indeed, all three subspecies appear as pleomorphic bloodstream parasites, having both long slender and short stumpy forms. However, molecular approaches have shown that this group is highly heterogeneous (33). The exclusive presence of the serum resistance associated (*SRA*) gene in *T. b. rhodesiense* has been used as a marker for the identification of this subspecies (34, 35). *T. b. gambiense* specific identification can be done through PCR amplification of the *T. b. gambiense*-specific glycoprotein (*TgsGP)* gene that encodes for a receptor-like glycoprotein, which is also involved in normal human serum resistance (36).

The third species within the subgenera of *Trypanozoon*, responsible for livestock infections, is *Trypanosoma equiperdum*. This parasite can be sexually transmitted amongst species from the family Equidae (horses and donkey), causing a venereal disease known as Dourine. Due to this transmission mode, the parasite has also acquired a wide geographic distribution. As for *T. evansi*, to which it is very closely related, also the classification of *T. equiperdum* as a separate species has been under scrutiny for many years (37), and several authors have suggested that there is no scientific argument to make a species level distinction between the two.

The second trypanosome subgenus responsible for salivarian trypanosomosis is *Dutonella*, which is mainly composed of two species, i.e., *T. vivax* and *T. uniforme*. Due to the global socio-economic impact caused by *T. vivax* infections in domestic animals, most of the studies of this subgenus have been carried out on this particular parasite species. This trypanosome is a pathogenic parasite species mainly found in Africa and South America (38). So far, only a single human infection has ever been reported (11). In Africa, transmission occurs in large through the bite of tsetse flies, having the highest infection rate of any tsetse-transmitted trypanosome species (39, 40). This high infectivity could be attributed to the relatively simple cycle of development in the vector's mouthparts. The transmission beyond Africa is mainly carried out mechanically by hematophagous flies from the genus *Stomoxys* and *Tabanus*, which transmit the disease to domestic animals such as cattle and goats, as well as to endemic wild animals such as capybaras, deer, and bubaline antelopes. Despite its large economic impact, especially in South America, *T. vivax* remains one of the less studied animal-infective *Trypanosoma* species. The main issue with *T. vivax* in the context of experimental parasitology and immunology research is the fact that virtually no parasites of this species are capable to be grown in mice. Hence, virtually all laboratory infections in mouse models (incl. Tv700, STIB 719, STIB 731-A, ILRAD 560, IL 1392) are being executed with the derivatives of Y468 *T. vivax* clone that was originally isolated in Nigeria that happened to “grow well” in mice after extensive adaptation (41). Hence, it remains to be seen if the TvY486 *T. vivax* reference strain is a good representative parasite for the general population of parasites found both in Africa and South America regarding host-parasite interaction mechanisms.

The third subgenus of pathogenic salivarian trypanosomes is *Nannomonas*, encompassing three species of animal-infective trypanosomes, i.e., *Trypanosoma simiae, Trypanosoma godfreyi*, and *Trypanosoma congolense*. The two first species are mainly infective to mammals belonging to the Suidae family (domestic pigs, warthogs etc.) while *T. congolense* has a broader range of hosts including livestock and game animals, but is generally accepted to be non-infective to humans. It should however be mentioned that a mixed *T. b. gambiense/T. congolense* infection has been reported in a human (42) and that *in vitro* testing of human serum-induced trypanolysis has shown a resistance phenotype in several stocks (43). *T. congolense* is the major tsetse transmitted pathogenic salivarian livestock trypanosome present in sub-Saharan Africa and causes large economical losses in the countries where it is endemic. The disease caused by this parasite is referred to as Nagana, meaning *depressed spirit* in the Zulu language of Southern Africa. During transmission, *T. congolense* develops in the midgut and proboscis of the tsetse vector. Mechanical transmission can occur, involving mainly *Tabanus* and *Stomoxys* species (44). In general, *T. congolense* is considered to be a monomorphic parasite. The host-parasite interaction as well as immunopathology associated with *T. congolense* infections has been better studied than in the case of *T. evansi* and *T. vivax*. However, it is important to point out the fact that while the molecular parasite surface structure has been well-described and compared to *T. brucei* (45), the regulation and kinetics of surface coat variation, as well as the infection of the coat with the immune system have never been analyzed in detail. Hence, most statements about these interactions and regulations are based on the assumption that *T. congolense* should behave the same way as *T. brucei*. A second scientific issue that plagues the *T. congolense* research literature is the fact that it is often used as a “chronic” model in comparison to “acute” *T. brucei* infections. This artifact originates from the fact that a specific chronic *T. congolense* clone, i.e., Tc13, has been used in major immunological investigations for almost three decades (46). In contrast, the vast majority of experimental host-parasite research in *T. brucei brucei* and *T. brucei rhodesiense* models has been done with much more virulent *T. b*. AnTat 1 or LouTat 1 clones (47–49). While these studies by themselves all resulted in valid experimental data, it should be said that there are virulent *T. congolense* strains, resulting in infections in mice which display very similar survival times as the *T. brucei* clones mentioned above (50). Unfortunately, these more virulent *T. congolense* isolates have not been systematically used in comparative studies with *T. brucei*. Hence, reports that compare highly virulent *T. brucei* infections with low virulent *T. congolense* infections, and subsequently provide conclusion in which infection outcome is linked to the species-specific background of the parasite, should be taken with utmost caution. Of note is that the high genetic heterogeneity of *T. congolense* has led to the division of this parasite in three different subgroups i.e., Savannah, Kilifi, and Forest within the same species (51).

## General life cycle of salivarian trypanosomes and interaction with both insect and mammalian hosts

**Figure 2:**
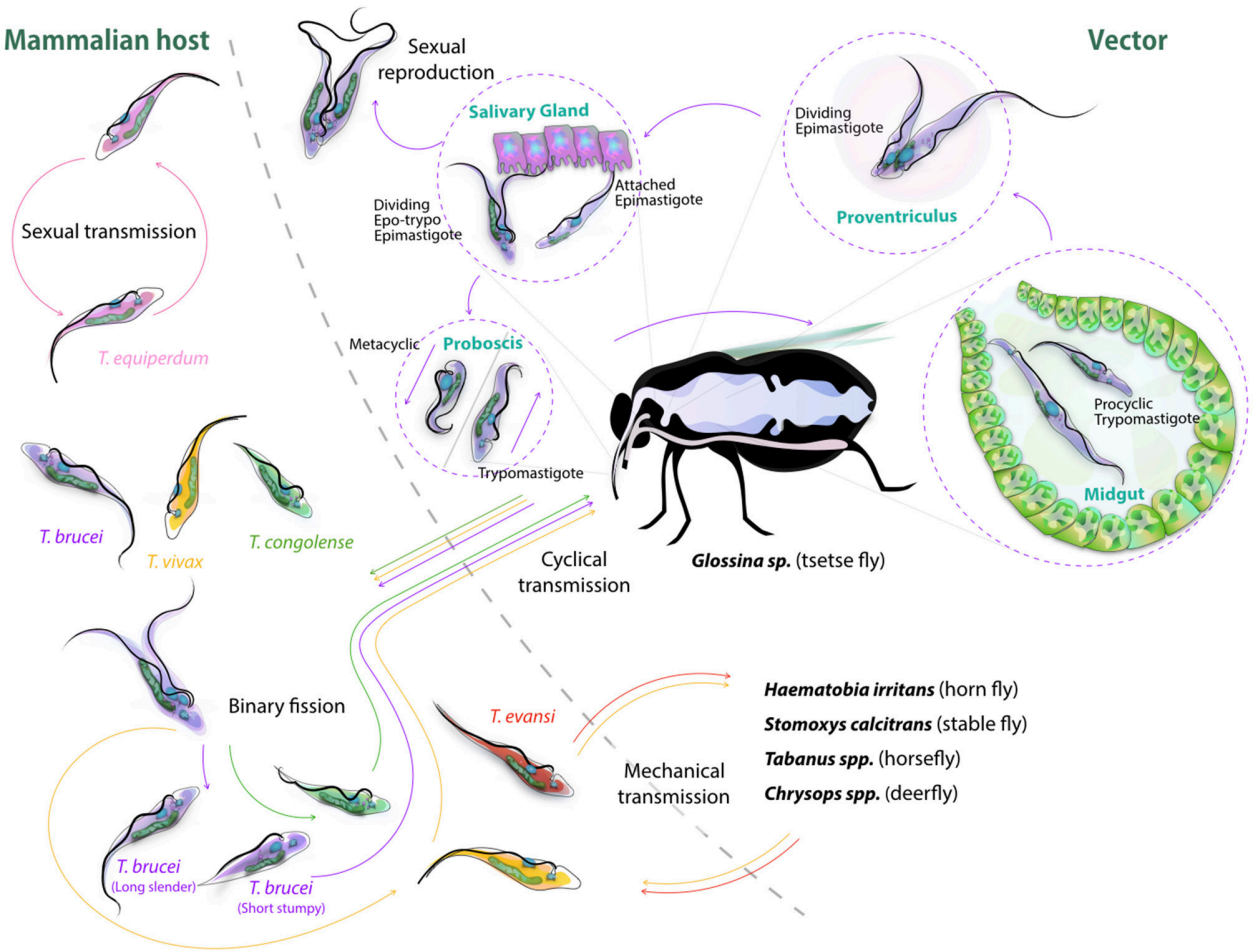
Cyclic transmission of salivarian trypanosomes mediated by tsetse and other biting flies. Most lifecycle data of African Trypanosomes is based on the *T. brucei* cycle involving the tsetse (52). Here, short stumpy form parasite are taken up by the fly, progress through the body of the insect vector passing via the midgut, proventiculus and salivary gland, to be re-injected though the proboscis as infective metacyclic trypomastigotes into a new target. In the bloodstream, differentiation to long slender forms occurs followed by binary fission proliferation. Differentiation back to short stumpy forms will complete the cycle. While *T. congolense* and *T. vivax* can follow a similar cyclic transmission mode, the latter also is known to be spread through mechanical transmission (53, 54), as is the case for *T. evansi* (1). *T. equiperdum* spreads through sexual transmission only (37). Sexual reproduction of trypanosomes itself has been reported to take part in the teste, and has been reported for both *T. brucei* and *T. congolense* (5).

The life cycle of cyclically transmitted salivarian trypanosomes inside their vector shows the plasticity of those parasites to adapt to new environments (Figure 2). Today, most of the knowledge on parasite transmission comes from the *T. brucei* model in which infection of tsetse flies occurs when the non-dividing short stumpy bloodstream form parasites are taken up during a fly's blood meal, reaching the fly's midgut and transforming into procyclic trypomastigotes (52). Once the parasite infection is established in the fly's midgut, the parasites migrate anteriorly to the proventriculus of the fly. Here, elongated trypomastigotes start to divide asymmetrically into both long and short epimastigotes of which the latter migrate toward the salivary glands and attach to the epithelial cells of the gland. Next, a final division occurs giving rise to the host-infective metacyclic forms. Once in the host's bloodstream, metacyclic parasites transform into long slender shaped bloodstream form parasites, which further divide by binary fission and represent the active dividing parasite form during the mammalian infection stage. It is at this stage that trypanosomes express the Variant Surface Glycoprotein (VSG) (55). This coat protein is encoded by a battery of over 1,000 different genes, mosaics and pseudogenes (56) and serves as an antibody decoy defense system (57). Indeed, VSGs are highly immunogenic and induce VSG-specific antibody responses. Hence, by regularly altering VSG variant expression, the parasite avoids efficient immune recognition and destruction (58). Genetic regulation of antigenic variation has been studied in detail and has been shown to involve various mechanisms of DNA recombination and transcription regulation of VSG genes (59, 60). With respect to mechanisms of genetic variation in trypanosomes, there is now ample evidence supporting the fact that *T. brucei* parasite mating or sexual reproduction does take place in the tsetse (5, 6). Hence, while the tsetse is conventionally referred as the insect vector for trypanosomosis, it should actually be considered as the definite host for the parasite. To put it in other words: mammals are merely the vessel that is used to ensure that trypanosomes are able to migrate from one tsetse to the next, and in addition provide long-term reservoirs that allow trypanosomes to survive seasonal periods in which fly populations are diminished. Sexual reproduction inside the insect vector offers the parasite in theory the chance of generating new hybrids, combining different parental characteristics. Important to note however is the fact that the effectiveness of trypanosome infection in the fly rapidly decreases with the age of the fly, hence also affecting the chance to generate hybrid descendants. Using both green and red fluorescent trypanosomes to study hybrid formation, it was shown that midgut and salivary gland infection rates were highest when flies were exposed to parasites in their first feed (53). Waiting 2^1/2^ weeks for a first parasite exposure reduced the infection success by half. Interestingly, exposing tsetse flies to two different trypanosome lines in a consecutive feeding experiment resulted most often in the establishment of the first infection only, as if the primary infection was able to push the vector to mount a protective immune response preventing secondary infection. Under natural circumstances, this would greatly reduce the chance of hybrids being formed, although the experimental conditions used above showed that in all combinations tested, hybrid formation did take place (53). As will be outlined later in this review, even the rarest hybrid formation events can have a significant impact on the transition from AAT to HAT, as it allows generation of a continuous pool of new human infective *T. rhodesiense* parasite strains (54). With respect to the vector immunity mentioned above, several studies published in the recent past have made contributions to the understanding of the mechanism underlying tsetse anti-trypanosome immunity. It is interesting to note that tsetse immunity development *per se* requires the presence of the obligate symbiont *Wigglesworthia* in the larval stage of the fly, transmitted through maternal milk gland secretion (61, 62). This finding complements the notion that the development of a fully functional innate immune system of the mature adult tsetse fly depends on the establishment of a bacterial microbiome population, and that the immaturity of the immune system is responsible for the high susceptibility to trypanosome infections during a first blood feeding (63). The fly immunity itself relies on multiple mechanisms. Indeed, the action of scavenger receptor peptidoglycan-recognition protein LB (PGRP-LP) is crucial for the colonization of the fly by its *Wigglesworthia* symbiont, and in addition has a direct trypanocidal activity on both procyclic and bloodstream form trypanosomes (64, 65). In addition, anti-trypanosome immunity relies on activation of the immune deficiency regulated pathway and antimicrobial peptides (66, 67), as well as reactive oxygen species (ROS) mediated defenses (68), which provides combined protective immunity at the level of the midgut and hemocoel. Interesting here is that for some time the peritrophic matrix, which is a chitinous protective layer lining the insect gut, has been considered as a physical barrier that could provide protection against invading infections. However, RNA interference-based reversed genetic approaches have shown that the matrix is a true immunological regulator. Its integrity is necessary to build a proper immune context in the defense against different microbes, including trypanosomes, through its role in the expression of the antimicrobial peptide attacin as well as dual oxidase and iNOS, both involved in the production of reactive oxygen intermediates (ROIs) (65). Finally, the tsetse fly's specific TsetseEP protein was shown to provide anti-trypanosome protection at the level of the midgut (69). Interestingly, starvation of flies reduces immune responsiveness and increases susceptibility toward trypanosome infections both in young and older flies (70–72).

Naturally, from a human and economic point of view, it is the mammalian infection stage that has attracted most attention in the past. It is only more recently that the parasite-vector interaction and biology has received more detailed attention and that also non-*T. brucei* infections have been studied in tsetse (73, 74). These reports show that in fact both *T. congolense* and *T. vivax* are much more effective in establishing tsetse infections than *T. brucei*. In particular, for *T. congolense* it has been shown that this parasite is particularly effective in reaching the proboscis of the fly, where the trypomastigote-epimastigote transformation takes place. In this case, migration from the foregut to the mouthparts appeared to occur with high efficiency. In contrast, *T. brucei* is much less efficient in colonizing the tsetse, as most parasites do not survive the migration from the foregut to the salivary glands. Investigating both *T. brucei* and *T. congolense* infections in parallel have suggested that *T. brucei* adopted to final survival in the salivary gland, as this niche would not be preoccupied by the much more efficiently growing *T. congolense* parasites. Hence, despite the fact that both parasites use the same transmission vector, and that also for *T. congolense* meiotic reproduction has been reported in the teste vector (75), there are remarkable differences in the way the two trypanosomes infect and occupy the body of the insect host. In recent years, specific attention has also been given to the immunological events that take place at the bite site of the tsetse, in order to explain how successful mammalian infections are initiated. Here, it has become clear that there is a crucial role for tsetse saliva components in preventing local blood clothing, vasodilation, and neutrophil influx, all leading to the successful establishment of a primary infection and allowing metacyclic saliva parasites to be transformed successfully into long slender bloodstream form parasites (76, 77).

Besides the biological vector transmission described above, mechanical transmission is a second way of ensuring parasites can move from one mammalian host to the next. This mode has been described for *T. congolense, T. evansi*, and *T. vivax*, but only the latter two have successfully used this way of transmission to migrate out of Africa, into other continents. The main vectors that have been reported today for both trypanosome species are the horsefly, stable fly, horn fly, and deerfly. In all cases, mechanical transmission occurs when a fly with blood-contaminated mouthparts, containing living bloodstream form parasites, rapidly changes feeding hosts allowing the parasites to be transmitted without any intermediate insect-specific forms. To date, virtually no information is available on possible immune interactions that could make this way of parasite transmission more or less successful. Interestingly, also nothing is known about the immune events that aid in parasite transfer back to the fly. Indeed, it is remarkable that livestock parasitaemia levels are often extremely low, presenting blood parasite load levels that are hardly detectable by microscopy. Yet, even when circulating parasite numbers are extremely low, flies still manages to successfully pick up parasites while taking very small blood meal volumes. Whether this is due to the fact that parasitaemia in the skin microvasculature is uniquely high as compared to the general blood circulation, or whether fly saliva has unique and potent trypanosome chemoattractant, remains to be elucidated.

## Human trypanosomosis in Africa is only part of the problem

In 2009, the number of reported HAT cases dropped below 10.000 for the first time in 50 years, and the most recent figures available for 2015 indicate that the global incidence of HAT has dropped below 3.000. It is now estimated that by 2020 HAT will no longer be considered as a major human health problem in Africa and hence will also no longer be listed as a neglected disease (78). With HAT being caused by either *Trypanosoma brucei gambiense* (>97%) or *Trypanosoma brucei rhodesiense* (<3%) (79), these numbers and assumptions are mainly based on the current West/Central African situation. Considering however that *T. b. rhodesiense* is a zoonotic parasite with a cattle and wildlife reservoir, re-emergence of HAT is going to remain a crucial concern in Africa. Indeed, whether or not the reservoir of *T. b rhodesiense* has been brought under control is hard to verify due to the insufficient systemic reporting on *T. b. rhodesiense* game and cattle infections. In addition, beyond the borders of the African continent the existence of non-*T. brucei* human trypanosomosis could become a future problem as *T. evansi* infections spread around the globe. Despite the reports of *T. evansi* infections in humans (80–83), this parasite is still not widely considered as a human pathogen. The lack of interest in these infections, combined with the continued spread of these trypanosomes mainly in South America, the Indian subcontinent, and Asia, risks of exposing humans to a new type of “unconventional” disease that will require a whole new approach to trypanosomosis world-wide. In addition, the lack of experimental studies on *T. evansi* infections as compared to *T. brucei* infections makes it harder to link the discussion about the genetic classification of *T. evansi* as a *T. brucei* subspecies to data dealing with the cellular and molecular mechanisms that govern host-pathogen interactions, pathology development, and zoonotic behavior.

Taken the importance of the zoonotic aspect of most remaining human trypanosome infections, it is clear that (African) Animal Trypanosomosis (AAT) *per se* deserves particular attention. In fact, there is a wealth of information available with respect to geographic distribution of AAT, immune pathologies including anemia and vaccine efforts as well as failures. These reports outnumber the scientific data published on host-pathogen aspect of human trypanosomosis. However, this contrasts very much the situation with respect to the actual understanding of trypanosome diseases. Indeed, while many have focused on experimental models to understand human trypanosomosis in the past, much less effort has been made to understand the fundamentals of animal trypanosomosis. A fine example to illustrate this is the issue of antigenic variation. For more than four decades, scientists have been studying antigenic variation of the VSG coat used by trypanosomes to deliver a protective response against the host antibody defense system. All work that included genetic approaches as well as molecular biology approaches combined with computer modeling, has been done for *T. brucei*, largely ignoring the fact that the main cattle parasites i.e., *T. congolense* and *T. vivax* are very different trypanosomes that might use other systems of regulation and defense. For example, comparative studies of the VSG repertoire conducted in *T. congolense* and *T. vivax* indicated that the scale of the VSG recombination differs between two species, being more frequent in *T. congolense* than in *T. vivax*. Moreover, *T. vivax* populations were shown to be consistent with clonal reproduction (84), while the *T. congolense* ability to undergo sexual reproduction generates opportunities for allelic recombination among VSG genes (85). Hence, to date we are left with molecular tools that are very suitable to study a wide range of host-parasite interactions in mice which could model human infections, but at the same time we have very little experimental models and tools to study AT. One striking example is *T. equiperdum, which* is hardly studied in comparison to other infections due to the fact that it causes a disease characterized by sexual transmission in horses, a feature so far not reported in mice. In fact, only a single report is available on experimental sexual transmission of trypanosomosis in mice, and this report is dealing with *T. b. gambiense* (86). However, whether sexual transmission of *T. brucei gambiense* is a real issue in human trypanosomosis, remains a matter of debate (87) and more species-specific research is needed to resolve these issues.

## Adaptations of animal infective trypanosomes to human host

Humans are considered naturally resistant to pathogenic animal trypanosomes such as *T. b. brucei, T. evansi, T. equiperdum, T. congolense*, and *T. vivax*. The mechanism of human resistance to those trypanosomes is based on the presence of cytolytic factors in the high density lipoprotein (HDL) fraction of normal human serum (NHS) (88) and is attributed to two fractions called trypanosome lytic factor 1 and 2 (TLF1 and TLF2), with the latter being the major active compound (89). The human infective *T. brucei* subspecies *T. b. gambiense* and *T. b. rhodesiense* are known to be resistant to TLF lysis through various mechanisms. A number of reports demonstrating *T. evansi* infections in humans has triggered a new wave of interest in molecular mechanisms underlying human infectivity in the context of the transition from an animal infective trypanosome to a zoonotic pathogen causing disease in humans. While TLF1 and TLF2 have slightly different compositions, they both contain the haptoglobin related protein (HPR) and Apolipoprotein L1 (ApoL1), of which the latter is considered the main active lytic compound. Furthermore, TLF2 is complexed with IgM molecules and has a much higher molecular mass than TLF1, but is lipid poor (90). With respect to the mechanisms of lysis and resistance to NHS, initial data was obtained in an experimental model for *T. b. rhodesiense*. Here, it was shown that resistant and susceptible sub-clones can be derived from a common ancestor by passaging trypanosomes in either the presence or absence of human serum. This work gave rise to the discovery of the *SRA* gene, encoding a truncated VSG and located within the VSG expression site (91). Transfer of the gene to *T. b. brucei* showed that the presence of this single gene indeed was enough to confer resistance to NHS (92). Subsequently, the identity of SRA allowed to characterize ApoL1 as the active serum compound (93) that acts through both lysosomal and mitochondrial membrane permeabilization (94). The inhibitory mechanism of SRA was shown to rely on its capacity to block the membrane pore-forming capacity of ApoL1 upon entering the acid compartments of the lysosomal system (95–98). Finally, the haptoglobin-hemoglobin receptor (HpHbR), localized in the flagellar pocket, was identified as the trypanosome receptor involved in TLF1 uptake (99), while the mechanism for TLF2 uptake has still not been elucidated (100). Indeed, HpHbR knock down *T. b. brucei* parasites remain sensitive to both NHS and TLF2 lysis (101). However, as both TLFs contain the same active ApoL1 compound, the SRA is capable of blocking all NHS activity. Interestingly, the *SRA* gene is only present in *T. b. rhodesiense* and hence has been used in a string of different diagnostic approaches for HAT, including PCR and LAMP, targeting not just human samples but also livestock and game reservoirs as well as the tsetse vector, in various regions in Eastern Africa. Of note is a report in which a Ugandan HAT survey indicated that 20% of parasitologicaly confirmed *T. b. rhodesiense* cases resulted from infections that could not be detected by any of the *SRA* PCR methods described so far. Whether this indicates that *SRA*-negative human infective *T. b. rhodesiense* parasites exist, implying these parasites have alternative NHS resistance mechanisms, or whether the negative results were due to *SRA* gene polymorphism that prevented PCR primer annealing, remains a matter of debate (102). The existence of such *SRA* gene polymorphisms has been studied in the context of human disease severity (54). Most recently, using *SRA* as a genetic tracer, it has been shown that genetic recombination between *T. b rhodesiense* and the much larger pool of *T. b. brucei* animal trypanosomes allows for the continuous generation of new *SRA*-positive human infective parasites (103, 104). This once again indicates that in order to really bring HAT under control, AAT and particularly the animal *T. b. rhodesiense* reservoir has to be eliminated as well.

Interestingly, more than 97% of all human trypanosomosis infections are caused by *SRA*-negative *T. b. gambiense* (79), a parasite that is subdivided in Group 1 and Group 2 *T. b. gambiense* (105). While Group 1 parasites have an invariant phenotype toward NHS resistance, Group 2 parasites show a variable degree of resistance, modulated by the actual exposure to NHS. Neither Group 1, nor Group 2 parasites have the *SRA* gene (8), indicating that there are multiple ways to mount resistance to NHS. Literature of the last 5 years has mainly focused on the Group 1 *T. b. gambiense* resistance, showing that these parasites have acquired a combination of defense systems which allows them to resist the lytic action of NHS. This includes a specific point mutation in the HpHbR in combination with reduced receptor expression, reducing TLF1 uptake (106, 107). However, as this receptor is not a main player in TLF2 uptake, *T. b. gambiense* needs additional mechanisms to survive in human blood. These involve the alteration of lipid membrane fluidity by the *T. b. gambiense*-specific glycoprotein (TgsGP) (108, 109), as well as increased cysteine protease activity in the digestive vacuoles of the parasite. The latter is believed to directly affect ApoL1 activity (110). Today, it remains unclear how *T. b. gambiense* Group 2 parasites deal with the toxicity of NHS.

*T. evansi* parasites express neither SRA, nor TgsGP, but now have been reported in several instances as human pathogens causing a-HT (11, 18, 80, 82, 111). Similarly to *T. b. rhodesiense* and *T. b. gambiense* Group 2 parasites, a remarkable phenotypic plasticity of *T. evansi* has been described upon exposure to NHS, with resistance occurring after prolonged NHS exposure and being absent when NHS selective pressure is removed. Screenings of various *T. evansi* isolates indicated that some even display natural resistance to NHS, while others were found to be fully sensitive (112). In line with these observations in *T. evansi*, even *T. b. brucei* NHS resistance has now been reported. In the latter, a switch to a resistant phenotype was recorded to occur upon repetitive exposure to NHS or TLF1 in the absence of the *SRA* gene (113). However, a fragment homolog to *SRA*, named SRA basic copy (*SRAbc*), was found in the *T. brucei brucei* TREU927/4 strain, which exhibits low resistance to NHS (114). Similarly, one of the human infective *T. evansi* isolates was shown to contain the *SRAbc* homolog (115). Interestingly, the *T. b. brucei* parasites exhibiting increased NHS resistance had a significant reduction in TLF1 uptake, which coincided with downregulation of *T. b. brucei HpHbR* mRNA levels (100). Hence, it seems that the resistance mechanism in these parasites shows a mixed but attenuated phenotype of those found in either *T. b. gambiense* or *T. b. rhodesiense*, as if the latter could have been selected as “optimized” derivatives of *T. b. brucei* semi-resistant predecessors.

With respect to human *T. evansi*, it is important to highlight that at least one human infective case has been attributed to the lack of functional ApoL1 (81). Indeed, a frameshift mutation, found in both *ApoL1* alleles of the patient, resulted in the ability of trypanosomes to establish infection and to survive in the human bloodstream. This strongly suggests that human trypanosome resistance in large relies on a non-classical immune mechanism, i.e., lipid membrane disruption by TLF. However, a Vietnamese a-HT *T. evansi* victim was shown to have fully functional *ApoL1* alleles and a normal concentration of serum ApoL1 (116). Hence, this shows that there is an additional role for the immune system in the overall defense against trypanosomes, most likely involving a combination of the action of antibodies, cytokines, and complement factors. In at least two confirmed human *T. evansi* infections, the infected individuals were shown to have a compromised immune system. One case relates to a pregnant woman from Mumbai, India suffering from HIV/AIDS, anemia, and upper respiratory tract infection (117). The second case relates to the above mentioned Vietnamese woman who had just given birth (116), with pregnancy itself being known as a unique immune condition that is modulated by fetus development resulting in immune alterations that in some cases in facilitation of parasite growth. Important is that also in experimental *T. evansi* infections in mice, in particular IgM antibodies are crucial for parasitaemia control (118). This indicates that when “natural resistance” such as the resistance conferred by ApoL1 fails, the antibody-mediated immune response does provide a second defense barrier against the progressing of infection.

Finally, it should also be mentioned that several reports in the past have indicated the existence of a-HT caused by the stercorarian *T. lewisi* parasite (119). Here, resistance to NHS lysis was correlated with resistance to human ApoL1 as well. Hence, it seems that while multiple mechanisms have been acquired by various trypanosomes to block the lysosomal pore-forming catalytic activity of NHS, this activity itself is executed in large by a single factor, i.e., ApoL1. This finding itself has attracted scientific attention over the last years with respect to primate evolution (120), and has resulted in the findings that (i) ApoL1 is the common lytic factor in human, gorilla, and baboon primate sera (7, 8, 121), (ii) the chimpanzee, orangutan, and macaque, which are susceptible to all *T. brucei* subspecies, lack functional ApoL1 (121), and (iii) the baboon ApoL1 variant is capable of killing even *T. b. gambiense* and *T. b. rhodesiense*, as opposed to the human ApoL1 (122, 123). The latter finding has prompted an attempt to generate genetically modified TLF transgenic livestock that would be able to resist all known pathogenic trypanosome species (122).

## The role of B cells and antibodies in salivarian trypanosomosis

**Figure 3:**
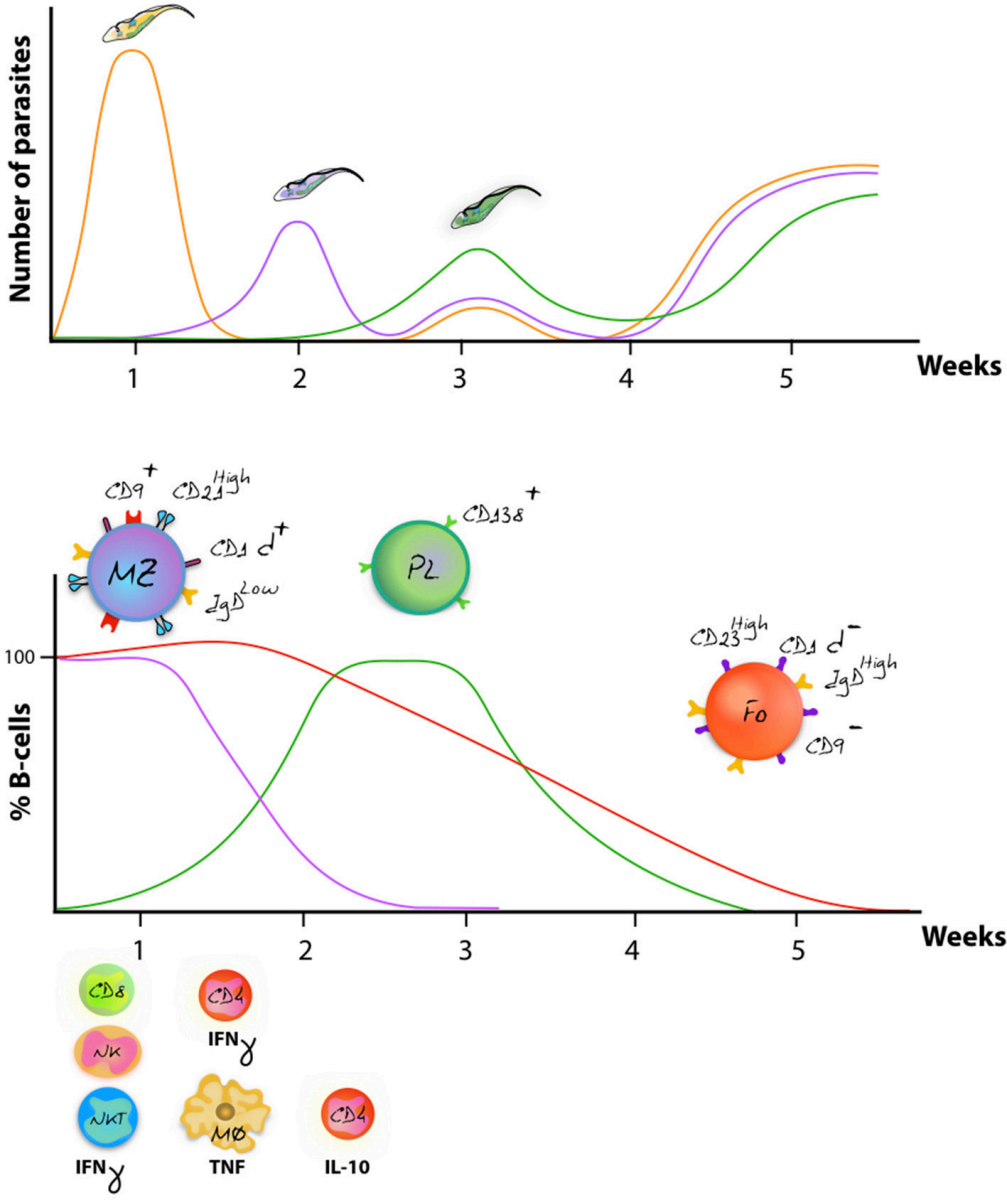
Antigenic variation and host immune destruction are closely linked. Antigenic variation of the trypanosome VSG surface coat enables trypanosomes to escape specific antibody-mediated destruction, resulting in immunologicaly distinct parasites occurring at regular intervals (upper panel). To prevent total eradication, trypanosomes undermine the immune system by ablation of the B cell compartment. In mice, this results in abrogation of an efficient antibody mediated immune defense system, allowing different parasite variants to occur simultaneously (schematically represented as the week 3 situation). Despite to co-occurrence of several variants, later-stage parasitaemia peaks usually have a reduced magnitude in terms of actual parasite numbers as various non-B cell defense systems aid in parasiteamia control [upper panel, adapted from (134)]. The lower panel schematically represent the finding that onset of infection is followed by a rapid depletion of the MZ B cell compartment (purple), followed by a gradual destruction of the FoB cell compartment (red) (116, 119). While the initially host immune response generates effector Plasma B cells, later waves of newly arising parasite variants fail to be efficiently depleted due to the impaired capacity of the host to deliver a renewed Plasma B cell response (green). Overall immunopathology is initiated by excessive production of IFNγ during the first week of infection, involving mainly CD8^+^ T cells, NK cells, and NKT cells. By 7 days post infection, IFNγ production is taken over by CD4^+^ T cells, while activated macrophages now produce excessive amounts of TNF that contribute to pathology (135, 136). Later-on in infection, production of IL-10 has been documented to counteract the initial inflammation (137).

As outlined above, VSG switching and antigenic variation, including the generation of VSG mosaic genes, have generally be considered as the major defense systems that parasites have developed against the host's adaptive immune system (56, 124, 125). However, in recent years it has become clear that trypanosomes have developed several precautionary adaptations that provide a rescue in case they do get recognized by antibodies. The reason for this is obvious: even if different VSG variants exhibit different hypervariable loops, and even if mosaic VSG present new epitopes to the immune system, there is no reason to assume that the overall pool of infection-induced antibodies is not at all capable of detecting new parasite variants. In fact, the existence of the cross-reactive nature of anti-VSG antibodies has been used since the beginning of trypanosome immunology research. Here, the VSG of the first arising variant or living cloned parasites, expressing a single VSG, have been used to monitor fluctuating anti-VSG titers throughout infection for weeks or months (57, 126). More interestingly maybe is the fact that reinfection models have shown that weeks into a primary infection, mice can be killed by a secondary infection with virulent trypanosomes expressing exactly the same VSG as the primary infection (127). In order to understand these results, three major host-parasite interactions mechanisms have to be considered. First, trypanosomes have developed a very efficient way of driving endocytosis. This system allows to continuously clear surface bound antibodies from the VSG coat, preventing antibody-mediated lysis (128). Secondly, while antibody/complement-mediated lysis has long been proposed to result in antibody-mediated trypanosome killing, it is important to note that AKR mice, which are natural C5 complement knockout mice, are able to clear peaks of parasitaemia in a similar way as other mice. This shows that the lack of complement-mediated lysis does not prevent the immune system of controlling peak stage parasite levels (129). Third, there is now ample evidence that trypanosomes cause a B cell depletion pathology, which is initiated by the very rapid disappearance of immature B cells in the bone marrow, as well as transitional and IgM^+^ marginal zone B cells from the spleen, followed by a gradual depletion of Follicular B cells (FoB) (127, 130). This has now been reported for *T. b. gambiense* and *T. congolense* infections (50, 131). Mechanisms involved in the depletion process have been linked to IFNγ-mediated inflammation (132), NK-mediated B cell destruction (133), and direct cell-cell contact-mediated B cell apoptosis (111, 127). Once FoB cell depletion is accomplished by the parasite, it becomes impossible for the host to generate new efficient antibody responses against newly arising VSG variants (Figure 3). In addition, it hampers anti-VSG memory recall responses against previously encountered variants, hence making the host susceptible to secondary infections with old variants. This might also explain the accumulation of mosaic VSG variants during later stages of infection (56). Indeed, the question whether later variants are immunologically distinct (or not) from their ancestral variants, which has so far not been answered, might not be important at all. Possibly, these variants arise simply from the continuous gene rearrangements that are ongoing at the telemoric ends of the VSG expression sites and are tolerated, due to the lack of antibody-mediated elimination by the host, rather than being produced in order to evade the already existing antibodies.

**Figure 4:**
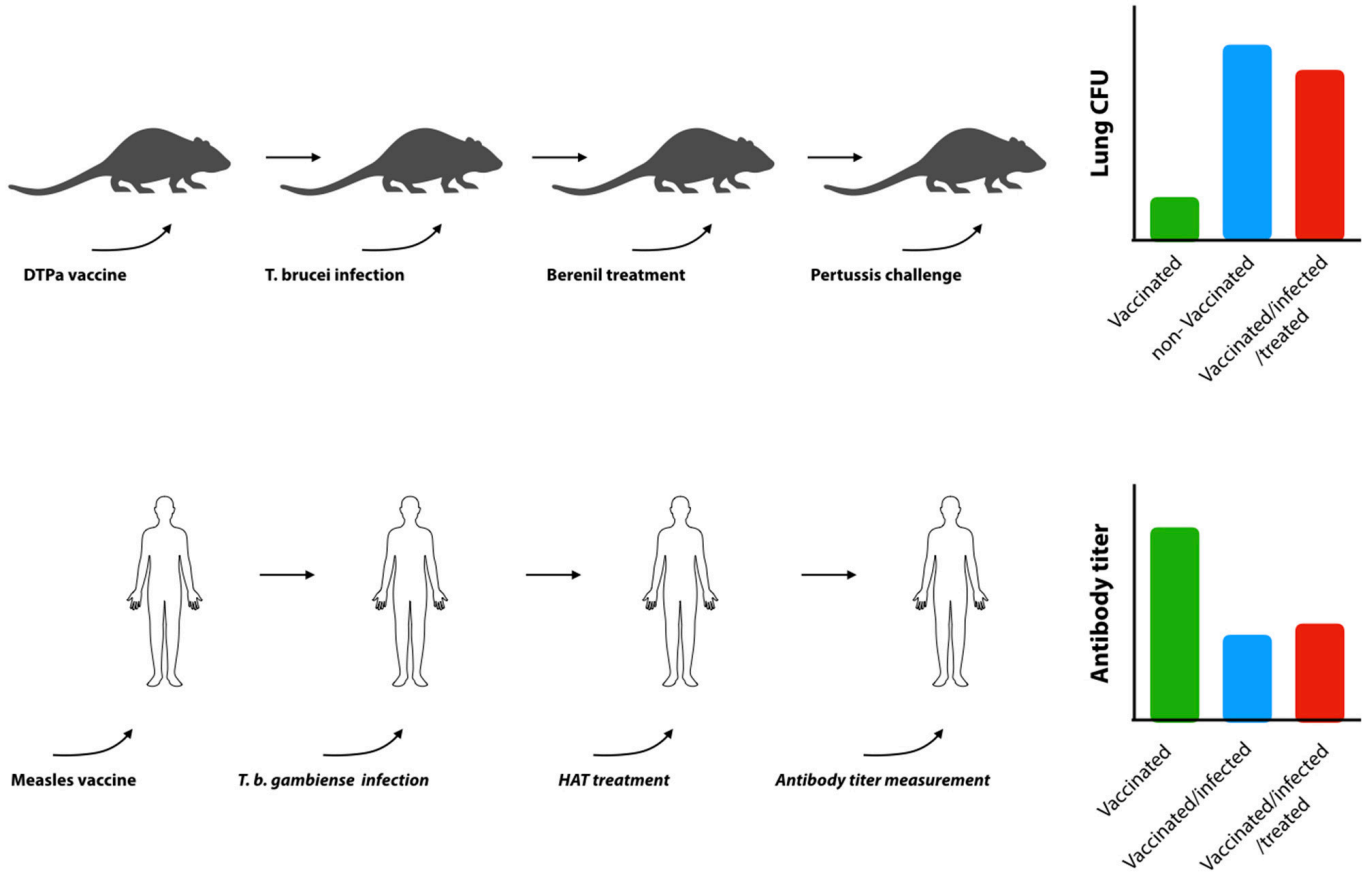
Trypanosomosis-induced B cell destruction results in prolonged B cell dysfunction. Experimental infections in mice have shown that trypanosomes can destroy non-related DTPa induced vaccine responses (upper panel). Vaccinated mice that have been confronted with trypanosomes fail to clear diphtheria bacteria from their lungs, even when the bacterial challenge is performed after the trypanosome infection has been cleared by drug treatment [adapted from (119)]. Indeed is was shown that while the commercial vaccine Boostrix® provides significant protection against a *B. pertussis* challenge (green bar: vaccinated, blue bar: non-vaccinated), exposure to trypanosomes abrogated vaccine-induced protection (red bar). In humans (lower panel), trypanosome infections were shown to suppress vaccine induced anti-measles antibodies. Serum antibody titers in vaccinated *T. gambiense* HAT patients (blue bar) were shown to be significantly lower as compared to vaccinated control individuals (green bar), and specific antibody titers did not recover after curative anti-HAT treatment (red bar) (121).

B cell dysfunction, associated to trypanosomosis, also has a secondary detrimental effect on the mammalian host, i.e., the elimination of vaccine-induced memory recall responses. Indeed, in an experimental model for DTPa vaccination, it was shown that *T. b. brucei* is capable of destroying immunological memory rendering vaccinated mice susceptible to infections with *Bordetella pertussis* (130). This was not a result of an infection-associated immunosuppression, as it persisted after anti-trypanosome drug treatment and the elimination of active trypanosome infections. This detrimental effect of trypanosomes on non-related vaccine efficacy has also been reported in other models and natural infections. Although not thoroughly studied in human infections, one study has shown that antibody titers induced by the anti-measles vaccine are significantly downregulated in HAT patients, and that curative HAT treatment did not result in a restauration of antibody titers (Figure 4). For obvious ethical reasons, this study stopped short of assessing whether or not the remaining titers would still confer protection. In addition, the study did not address the question whether vaccine-induced memory recall responses were affected (138). With respect to AT, more data is available in particular with respect to *T. evansi* and *T. congolense* infections. Indeed, such infections in pigs were shown to abrogate protective immune responses generated against the classical swine fever vaccine (139). They were characterized by significantly reduced antibody responses, leukopenia, and high fever. Similarly, *T. evansi* infections in water buffalos, vaccinated against *Pasteurella multocida* (hemorrhagic septicemia), showed impaired capacity to mount a humoral and cell-mediated immune response upon challenge (140). When cattle, harboring *T. congolense* and *T. vivax*, were given a *Brucella abortus* vaccine or were vaccinated against contagious bovine pleuropneumonia, specific antibody responses to the vaccine were shown to be severely depressed (135, 141–143). Similarly, *T. congolense* infected goats vaccinated with *Bacillus anthracis* showed a profoundly diminished anti-anthrax antibody responses (144), while *T. congolense* infected cattle were shown to suffer from immunosuppression and failed recall responses in a foot-and-mouth vaccination setting (145). Taken together, these studies, conducted in natural hosts for animal trypanosomes such as cattle, water buffalos, goats, and pigs, confirmed studies in mice showing that trypanosome infection induces severe impairment of B cell responses and antibody production to a number of non-trypanosome related commercial veterinary vaccines.

## The role of T cells and T cell-derived cytokines in salivarian trypanosomosis

Taken the extracellular nature of salivarian trypanosome infections, initial thoughts on the control of infection were naturally focused on the role of antibodies and B cells. However, already early in the 1980's it became obvious that while the virulence of experimental trypanosomosis was not linked to the expression of a specific VSG variant, or the use of a specific MHC-II type, CD4^+^ T cells played an absolutely crucial role in infection control (146). Twenty-five years after this initial discovery, it was shown that major T cell responses against cryptic T cell epitopes play a major role in trypanosomosis control (147, 148). This observation caused a major paradigm shift in the way trypanosomosis control is thought to occur, as it shows that T cell help is not just needed to support effective B cell functioning and antibody production, but that it plays a crucial second B cell-independent role during the progression of infection. This role of T cell biology might initially have been underestimated, as a multitude of studies had shown that trypanosomosis, both in mice and cattle, results in the occurrence of T cell immunosuppression (49, 149). However, these reports mostly referred to suppression of T cell proliferation and not to cytokine secretion. To date, the active disease controlling role of T cells is mainly attributed to the cytokine production in which polarization toward a Th1-type response is crucial for initial parasitaemia control (137). Detailed analysis of both *T. brucei* and *T. congolense* infections has indeed shown that early IFNγ production is crucial for the control of the onset of infection (Figure 3). This hypothesis was initially driven by the description of the cytokine production profile of CD4^+^T cells, and was later corroborated by the use of neutralizing anti-IFNγ antibodies as well as the use of IFNγ knockout mice (150, 151). The latter were shown to have an impaired control of the first peak of parasitaemia, followed by their inability to clear increasing parasite numbers, leading to the early death of the mice using the C57Bl/6 model (152). In experimental *T. congolense* infections, it was demonstrated that while hyper-susceptible BALB/c mice preferentially mount an infection associated Th2-type response against the Tc13 *T. congolense* parasite, Th1-biased C57Bl/6 mice were able to survive for up to 6 months when infected with the same clone (136). Also here it was shown that altering immune balances, by treatment with neutralizing anti-cytokine (or cytokine receptor) monoclonal antibodies, drastically alters the outcome of infection (153). Most recently, it was reported for *T. brucei* that the CD4^+^ IFNγ response is preceded by the production of this cytokine by NK and NKT cells, followed by a marked upregulation of IFNγ production by CD8^+^ T cells (154). This was observed in both the spleen and the liver of infected mice. However, by 10 days post infection it was clear that these cell populations were either drastically reduced in numbers or became totally depleted, leaving the CD4^+^ T cells to execute the major task of cytokine production. A comprehensive report on the overall role of IFNγ in various trypanosome infection models was recently published by Wu *et al*. (155). Interestingly, to date only one single trypanosome factor has been identified as being able to induce IFNγ production by T cells, CD8^+^ T cells in particular. This molecule, named TLTF (trypanosome lymphocyte triggering factor) (10) has been characterized in *T. brucei* and *T. evansi* parasites (156), but a homolog has not been described for other trypanosomes. TLTF was shown to be capable of inducing IFNγ production by astrocytes, suggesting a direct role of the molecule in the pathology development of sleeping sickness (157). This hypothesis has been further supported by the finding that IFNγ deficient mice show reduced CD4^+^ and CD8^+^ T cell influx into the brain parenchyma of *T. brucei* infected mice (158). Important is that when the role of T cells and IFNγ are considered within a trypanosomosis context, IL-10 was shown to be the main counter regulator of infection-associated inflammation in both *T. brucei* and *T. congolense* models. The source of the latter was proposed to be the regulatory T cells (9). However, a most recent effort to understand the full kinetics of IL-10 production during trypanosomosis, using the IL-10 reporter mouse system, has indicated that a whole range of cells is capable of producing IL-10 during infection and that most notably CD4^+^ T cells, that do not have a defined regulatory phenotype, are the main producers of this cytokine (159).

As for the role of IFNγ in parasitaemia control, early reports characterized this cytokine as a trypanosome growth factor (160). In contrast, a number of follow-up reports have indicated that IFNγ is crucial for inhibition of trypanosome growth, as well as for trypanosomosis-associated NO production and TNF production that both limit trypanosome growth (152, 161, 162). Both these factors have subsequently been identified as direct players in the control of peak parasitaemia levels in *T. brucei* as well as *T. congolense* infections. Using TNF knockout mice, it was shown that parasitaemia control in relatively resistant C57Bl/6 mice depends on TNF production during the first peak of parasitaemia (163, 164). This confirmed initial reports in which neutralizing anti-TNF antibodies had a negative effect on *T. brucei* parasitaemia control (165). Subsequently, a comparative infection model using BALB/c, C57Bl/6, C3H/HeN, and CBA/Ca mice showed that while TNF production *per se* was required for proper parasitaemia control, infected mice responded to TNF-associated inflammation with the shedding of TNF-R2 receptors. This, in turn resulted in limiting the TNF-mediated infection-associated immunopathology (166). Based on these results, and placing these observations in the wider context of trypanosome infections using various gene-deficient mouse models, it has been proposed that while soluble TNF could plays a pivotal role in parasitaemia control, it is the membrane-bound form of the cytokine that has a major impact on progressing inflammation and inducing pathology (167). When analyzing the possible direct effect of TNF on trypanosomes, results showed a differential outcome depending upon the model studied. First, it was reported for the *T. brucei brucei* AnTat 1.1 clone that TNF has a direct trypanolytic effect. The latter is mediated by a lectin binding domain of the TNF molecule that is located at the opposite site of the molecule as compared to the mammalian receptor binding site (168). Interestingly, the lectin specificity of TNF exhibits a high affinity for complex branched mannose sugars, such as those found on the trypanosome VSG molecule (169). Using a model for *T. brucei gambiense*, these findings were independently confirmed in an *in vitro* co-culture system (170). In contrast, experimental mouse studies with the human infective *T. brucei rhodesiense* LouTat 1 clone did not show a direct effect of TNF (171), suggesting that various trypanosome stocks can exhibit different levels of susceptibility to host cytokine-mediated growth regulation, as might be the case for IFNγ. Studies in trypanotolerant vs. trypanosusceptible cattle, using a *T. congolense* model, confirmed the possible role of TNF in parasitaemia control in natural infections (172), but here no link was made to a possible direct trypanolytic effect of the cytokine. Also, for the *T. congolense* infection mouse model, the direct action of TNF has not been reported but here TNF-R1 signaling has been associated to the combined release of soluble TNF and NO by activated macrophages. Those two combined were shown to play a crucial role in parasitaemia control, in conjunction with infection-induced anti-trypanosome antibodies (118). Interesting to note here is that in contrast to the limited knowledge on the molecular mechanisms of trypanosomosis-driven T cell IFNγ production, detailed research into the mechanisms of macrophage-derived TNF induction have been able to provide a more in-depth understanding of the host-parasite interplay. Indeed, first the VSG-GPI anchor was identified as the major trypanosome molecule responsible for TNF induction, with the trypanosome-specific galactose side-branch of the anchor playing a major role in macrophage activation (173, 174). Subsequently, it was shown that IFNγ could prime macrophages to become responsive to GPI-VSG and trypanosome stimulation (175). These results are in line with *in vivo* data showing that a rise in serum IFNγ values precedes the induction of infection-associated TNF (154). However, when macrophages are primed with VSG, prior to IFNγ, the cells are de-sensitize with respect to cytokine secretion (175). This may represent a way to prevent hyper-inflammation during the phase that corresponds to peak parasitaemia clearance, in which massive amounts of VSG are being released into the hosts' circulation. This information has been used to try and design an anti-disease vaccination approach for trypanosomosis (176). Although it was shown that repeated exposure to VSG-GPI prior to infection could indeed reduce the infection-associated pathology related to excessive macrophage activation, the results indicated that the vaccine approach did not result in the buildup of immunological memory involving antibodies or B cells. Instead, the reduction of inflammation, following the repeated GPI exposure related to an alteration of macrophage phenotypes, shifted the balance from infection-induced inflammatory type 1 cells to more anti-inflammatory type 2 cells or macrophages. This response was short-lived. Moreover, as the beneficial effect of the VSG-GPI treatment was observed to the same extent in both B cell deficient mice and in wild-type littermates, it became clear the approach could no longer be considered as a possible vaccine approach (177). In addition to the VSG-GPI, trypanosome DNA that contains high levels of unmethylated CpG sequences has been reported to be responsible for driving NF-κB an MAPK signaling pathways, resulting in the transcription of pro-inflammatory cytokine genes including *tnf*, in case of *T. brucei* infection(178). As for infection with *T. evansi*, the information on the role of IFNγ is limited, but data has shown that in addition to anti-trypanosome antibodies, this cytokine is required for parasitaemia control (179). Most recently, a comparative cytokine analysis using different *T. evansi* infections confirmed the induction of IFNγ in all infections (180). For *T. vivax* infections, there is a general lack of published information on the role of T cells and their cytokines in the control of onset in infection, but it appears reasonable to assume that a similar cytokine environment is needed to obtain the best parasitaemia control possible. As for human (*T. b. rhodesiense*) and cattle (*T. congolense*) infection, the role of IFNγ in inducing inflammatory pathology has been confirmed in both (181, 182), and as already outlined above, IFNγ has been shown to play a major role in the actual cerebral complications that characterize human sleeping sickness (183).

## Anemia is a major pathological feature of animal trypanosomosis

From the data reviewed above, it has become clear that the IFNγ/TNF driven immunopathology is a hallmark of trypanosomosis in both human and livestock. When focusing on the latter it has been amply documented that TNF-linked infection-associated anemia is the major pathological feature that marks both *T. congolense* and *T. vivax* trypanosomosis. In fact, the classification of so-called trypanotolerant and trypanosusceptible animals is based on the relative capacity to control anemia during infection which consequently is directly linked to whether or not animals remain productive while infected with trypanosomes (184). Mechanisms that underlie this tolerance are complex and to date still not clearly understood, but most likely include differences in erythropoeitic potential and hemodilution (185), factors involved in erythrolysis (186), eryhtrophagocytosis (187), the regulation of the erythropoietin homeostasis (188), the host's potential to raise neutralizing antibodies against secreted trypanosome virulence factors (189), and the general mechanism involved in inflammation control such as the regulation of the IFNγ/IL-10 balance as well as other cytokines (167). With respect to cytokine regulation during infection, it is worth mentioning that MIF (Macrophage migration Inhibitory Factor) was recently shown to be one of the key trypanosomosis-associated inflammation inducing cytokines involved in the promotion of so-called anemia of inflammation, in which classically activated macrophages also play a major role (190). Indeed, it was shown that MIF promotes the storage of iron in liver myeloid cells, subsequently depriving iron from the eythropoeitic system and preventing the maturation of RBCs. Important here is that the *T. congolense* C57Bl/6 mouse model has been proven useful over the years to unravel the molecular mechanisms that govern anemia pathology. Gene expression profiling studies in these mice have pinpointed infection-induced deregulation of erythropoiesis as well as the involvement of stress-induced acute phase responses linked to *T. congolense*-associated anemia (191). More recently, the C57Bl/6 model was also shown to be suitable for unraveling the pathology of *T. vivax* infections (129, 192, 193). In addition, the use of various knockout mouse strains generated on the C56Bl/6 background, such as TNF^−/−^ and Lymphytoxin^−/−^ (194), TNFp75^−/−^ (166), IFNγR^−/−^ (152), IL-10^−/−^ (195), and Gal3^−/−^ (196) have all been used to study specific aspects of the inflammatory cascade that drive trypanosomosis-associated inflammation. One of the most recent finding here has shown that the reduced PCV, associated with experimental mouse trypanosomosis, is not just the result of reduced circulating RBC numbers, but also of increased plasma volume, leading to hemodilution. The reduction of platelet concentrations in *T. congolense*-infected mice has also been reported in *T. congolense*-infected cattle and sheep (197, 198) suggesting that hemodilution could be a major pathogenic characteristic of livestock trypanosomosis, as well.

To date, the characteristics of anemia are probably the most and best described features of AT. Whenever new surveillance efforts result in the discovery of new infection foci, anemia is usually the parameter that is used to describe disease severity. This is the case for *T. evansi, T. congolense*, and *T. vivax* independently of the geographic location of infection or host species. As described for *T. congolense, T. evansi*-induced anemia was linked to infection-associated deregulation of the iron metabolism as well (199), although the number of detailed mechanistic studies available for this infection are rare. Interestingly, trypanosomosis-associated anemia has even been observed in more rare infection cases of wildlife in the Australian little red flying fox infected with *T. minasense* and *T. rangeli* (200). Together, all these reports indicate that the general occurrence of trypanosomosis-associated anemia is most likely due to the multitude of inflammatory pathways, and linked to various aspects of impaired RBC development and clearance. Taken this into account, it is very intriguing that reports of severe infection-associated anemia are missing in the case of HAT. Indeed, most reports documenting HAT-associated anemia describe the induction of RBC lysis and acute anemia as the result of treatment rather than the infection itself. This is particularly the case for *T. b. gambiense* infections (201–204). For *T. b. rhodesiense* rare reports do exist on the occurrence of infection-associated anemia and hemolysis (205), and models have been developed using vervet monkeys to investigate this aspect of acute HAT pathology in details (206). However, at the same time other reports have shown the lack of any correlation between *T. b. rhodesiense* severity in humans and the presence or absence of anemia (207–209). Hence, it seems that the human immune system might have found unique ways of dealing with trypanosomosis both in terms of dealing with parasite killing through the TLF1/2 ApoL1 mechanisms, as well as the control of infection-associated anemia.

## Conclusion

Over the past years, international joint efforts to control Human African Trypanosomosis have resulted in a drastic reduction in the number of confirmed disease cases. In addition, it might be feasible that by 2020 the incidence of this neglected tropic disease will be reduced to levels where it is no longer considered as a public health threat. However, at the same time the incidence of animal trypanosomosis is on the rise, affecting animal productivity through the detrimental health effects caused by excessive host-parasite immune interactions. This has now become a world-wide issue that affects agricultural infrastructures in emerging economy countries, not just in Africa but also in Asia and South America. In addition, AT even threatens the livestock industry of developed countries when sudden outbreaks occur due to importation of the disease either through infected vectors or host animals. To date, not a single vaccine strategy is available to control AT. Detrimental infection-associated mechanisms undermine both T cell and B cell compartments, making it virtually impossible to develop methods that allow the generation of long-term sustainable immunological memory. It appears that in the evolutionary battle between the trypanosome and the host immune system, the parasite has not only been able to find ways to evade innate trypanosome killing mechanisms, but has also acquired tools to undermine the crucial immunological defense and memory systems of the host. Only recently, it has become obvious that one of the major strengths of the African Trypanosomes' defense system is the capacity of genetic exchanges between individual parasites, while residing in the insect vector. Through this mechanism, new human-infective parasites can be generated and this by combining resistant genes from existing *T. b. rhodesiense* parasites with the vast repertoire of the *T. b. brucei* animal parasite reservoir. In addition, it has become clear that mechanisms of genetic plasticity also have allowed *T. b. brucei* to transform into *T. equiperdum* and *T. evansi*, parasites that have now successfully conquered large areas of the developing world, as they no longer need the tsetse vector for transmission. In particular, the latter risks to become a future health problem, as human infective cases of *T. evansi* have already been reported in India, Vietnam, Egypt, and Algeria. Together, these results show that while classic *T. gambiense* trypanosomosis is becoming rare, AT and new atypical human trypanosomosis remain a serious risk for the human population. Future research into the host-parasite interactions and trypanosomosis- associated inflammation of these new atypical infections should allow to obtain a better understanding of problems, including vaccine failure, and hopefully lead us to a long-term solution that can deal with both human and livestock infections, as well as the ever surviving wildlife trypanosome reservoir.

## Author contributions

All authors contributed to the writing of the paper. In addition all artwork was prepared by JP.

### Conflict of interest statement

The authors declare that the research was conducted in the absence of any commercial or financial relationships that could be construed as a potential conflict of interest.
